# Spa Treatment (Balneotherapy) for Fibromyalgia—A Qualitative-Narrative Review and a Historical Perspective

**DOI:** 10.1155/2013/638050

**Published:** 2013-07-31

**Authors:** Jacob N. Ablin, Winfried Häuser, Dan Buskila

**Affiliations:** ^1^Institute of Rheumatology, Tel Aviv Sourasky Medical Center and Sackler Faculty of Medicine, 6 Weizman Street, 64239 Tel Aviv, Israel; ^2^Department of Internal Medicine I, Klinikum Saarbrücken, 66119 Saarbrücken, Germany; ^3^Department of Psychosomatic Medicine, Technische Universität München, 81675 München, Germany; ^4^Ben Gurion University, Department of Medicine H, Soroka Medical Center and Faculty of Health Sciences, 84101 Beer Sheva, Israel

## Abstract

*Aim*. To perform a narrative review of spa therapy for management of the fibromyalgia syndrome (FMS), evaluating this traditional time-honored form of therapy in a historical perspective. Methods. Medline was searched using the terms “Spa therapy,” “Balneotherapy,” and “Fibromyalgia” between 1990 (year of ACR fibromyalgia criteria publication) and April 2013. The Cochrane database was also searched. Publications relating to the implementation of spa therapy and related practices over the centuries were identified through references, searched, and reviewed. *Results*. Reports of balneotherapy were described from diverse locations throughout Europe and Asia, and various forms of water-related therapy have been incorporated for many musculoskeletal indications. In the management of FMS, spa therapy has generally been shown to be well accepted and moderately effective for symptom reduction. *Conclusion*. While achieving high-quality evidence-based conclusions is difficult for complex natural therapies such as spa therapy, the existing evidence indicates a positive effect in management of FMS. In view of the long history of this modality in the management of rheumatic pain as well as the inherent difficulties related to pharmacological treatment, the role of spa therapy should currently be recognized as part of a therapeutic program for FMS.

## 1. Introduction

Hot water has been used for thousands of years and throughout far apart cultures and locations as a treatment for musculoskeletal aches and pains. Distinct sites endowed with unique physical and chemical qualities have attracted the attention of individuals suffering from a broad spectrum of problems traditionally clustered under the title of “rheumatism” as well as for social, ritual and recreational purposes. While the precise mechanisms underlying the capacity of warm water to sooth and heal remains incompletely understood, the mass and diversity of documentation regarding these forms of therapy seem to make it self-evident that therapeutic merit does exist. On the other hand, making evidence-based recommendations remains challenging in a field where treatments are so diverse and lacking in standardization. Fibromyalgia syndrome (FMS) is in many ways the prototype of “rheumatism”, a condition characterized by widespread musculoskeletal pain, fatigue, and malaise. Thus, the application of spa therapy (balneotherapy) to the treatment of FMS is a natural choice. Keeping in mind the scientific limitations regarding the assessment of spa therapy for FMS, we present a narrative-qualitative review of this topic, in an historical perspective. 

Water is the essence of life. Humans, like other life forms, are absolutely dependent on the constant availability of water, and human civilization has necessarily developed alongside fresh water sources. Not surprisingly, ever since antiquity, water has been given an important place in human culture, religion and recreation. Water has been ascribed the spiritual and moral aspect of cleansing (the body and presumably the soul as well) and as such has also been expected to possess the capacity to purify, correct, and cure. Thus, the application of water for medicinal purposes dates back to ancient times and is found in diverse and far-reaching cultures around the globe. In the current paper we have performed a qualitative-narrative review regarding the role of water-based therapy, particularly spa treatment (Balneotherapy) in a historical perspective, focusing on the up-to-date evidence regarding the efficacy of this mode of treatment for chronic pain and fibromyalgia. The aim of the current review is not to perform a meta-analysis of the literature, as has recently been performed by Guidelli et al. [1], but rather to place current evidence regarding the application of spa therapy for FMS within a broad historical and cultural perspective, as the continuation of an ancient tradition, common to both western as well as eastern culture, of treating musculoskeletal pain with water.

## 2. Methods

Medline was searched using the terms “Spa therapy,” “Balneotherapy,” “Fibromyalgia” between 1990 (year of ACR fibromyalgia criteria publication) and April 2013. In line with our goal of performing a narrative review, the search was not limited to randomized controlled trials and review articles were included. 

In addition, for the purpose of adding a historical perspective, publications relating to the implementation of spa therapy and related practices over the centuries were identified through references, searched, and reviewed.

The Cochrane database was also searched. Selected articles were retrieved as well as relevant references identified during the initial search. Balneotherapy has been included in the operational definition of complementary and alternative medicine for the Cochrane collaboration [2], and reviews regarding the use of this modality in conditions such as osteoarthritis [3] and rheumatoid arthritis [4] are included in that database.

## 3. Spa Therapy: History and Geography

The term “spa therapy” used in this review is not the only one applied throughout history to the medical use of bathing in water. The precise origin of the term “Spa” are not known, and the appealing acronym “Sanitas per aquas” (health through water) is only one of the possibilities [5]. Various alternative terms include “Balneotherapy”, “Hydrotherapy,” and “taking the waters” [6]. Spa therapy can also be considered to be part of a system of biologically based treatments, due to the implementation of naturally occurring minerals found in spring water, which are presumed to contribute to the therapeutic effects of this treatment. 

 In ancient Greece, public bathing places were considered sacred places and were typically dedicated to specific deities [7]. Greek mythology as well is rich in tales regarding spring waters, gods, and goddesses. Thus, for instance, the Goddess Artemis was a patron of springs and controlled the nymphs, who were considered patrons of spas [8]. Hercules would bath in the springs of Thermopiles in order to restore his strength after undergoing his ordeals while the legendary Centauri (half man half horse) would heal the wounds caused by the arrows of Hercules by bathing in the springs of Kaifa. None other than Hippocrates wrote in his medical encyclopedia a chapter on “winds, water and places”. In modern times as well, Greece is rich in spas of different mineral composition as well as different temperatures. These locations are used for the treatment of a variety of conditions, with an emphasis on psoriasis and related dermatological conditions. 

Heavily influenced by Greek culture, the Roman Empire was similarly charmed by the temptation of spa therapy. Spas were used as places of recuperation for wounded soldiers, but also as places of recreation [6]. Spa therapy in ancient Rome included application and immersion in water, as well as drinking large volumes of water. The cultural significance of the spas appeared to vary over time, sometimes focusing on the medical aspects and other times more on the recreational. At the same time, Roman troops built bathing centers far away from Rome, throughout the Empire. 

The twentieth century has witnessed further developments in the historical development of spa therapy, including the interaction between natural resources and manmade enterprises. One unusual such case is exemplified by the case of the blue lagoon in Iceland, situated on the Reykjanes peninsula [9]. At this location, geologically formed by a ridge of porous volcanic lava, a high level of geothermal activity takes place. This has been utilized for the Svartsengi geothermal power plant, which resulted in the discharge of warm saline fluid and the creation of an artificial lagoon known as the blue lagoon. Through classical serendipity, a worker at the plant suffering from psoriasis discovered that the water and white mud of the lagoon had a positive effect on his skin condition. The unique geothermal microbial composition of the waters of the blue lagoon [10] as well as the presence of specific bioactive molecules has been suggested as having unique salutary effects on human skin [11]. Due to these properties, and undoubtedly also in virtue of the dramatic beauty of the landscape, the location has become a thriving spa and a tourist attraction. 

In Germany, hundreds of spa centers are active and are under specific standards of governmental regulation [12]. Mud spas are used for the treatment of both musculoskeletal and dermatological disorders. Seaside resorts along the Baltic and North Sea are common; another specific form of therapy used in dermatology includes phototherapy (saltwater-UV therapy), in which a patient baths in high-concentration sodium chloride and then is exposed to UVB. PUVA-bath therapy is also available. 

### 3.1. Spa Therapy in Asia

Throughout history spa therapy has not been confined to the western hemisphere. Dating back to ancient times, this form of treatment has been used by far reaching cultures for similar purposes. Thus, for instance, in Korea, spa therapy has been documented for the treatment of arthritis since the Silla dynasty (57 BC–935 AD), and in 1458 king Sejong “the great” declared the Onyang spa to be holy, after curing his chronic skin disease [13]. In Israel as well, a number of natural hot springs have been used for medical purposes dating back to ancient times [14], and the dead sea, which is a unique geological location, has been a special center of interest from this standpoint. 

## 4. Spa Therapy in Rheumatic Disorders

As previously noted, spa therapy of various sorts has been extensively applied for the treatment of painful conditions, with an emphasis on rheumatic disorders. Osteoarthritis, gout, rheumatoid arthritis, ankylosing spondylitis, and psoriatic arthritis are a partial list of rheumatological disorders which have been treated with these modalities [15]. The mechanism of action of spa therapy in the treatment of pain is complex and only partially understood. Water immersion has been shown to cause an increase in the levels of circulating opioid peptides [16] and has the capacity to reduce and relax muscle tone leading to an analgesic effect [17, 18]. The diuretic and natriuretic effect of water immersion has also been proposed as a mechanism of reducing joint swelling and decreasing pain [19, 20]. Bathing in mineral water may have specific effects dependent on the chemical composition of the water. Prostaglandin and leukotriene levels may be another target influenced by hot mineral water and mud packing [21]. Spa therapy may also act through inducing mental relaxation, reducing anxiety, and improving depression.  Table 1 summarizes examples of applications of Spa therapy over historical and geographical locations. 

## 5. Spa Therapy for the Fibromyalgia Syndrome (FMS)

In view of its long history in the treatment of musculoskeletal conditions and in the alleviation of pain in particular, it is not surprising that spa therapy has attracted particular attention for the management of FMS. This common condition, which is characterized by the presence of chronic widespread pain and tenderness, is considered to represent the clinical manifestation of central nervous system sensitization [22]. Although some progress has been made in the development of pharmacological treatments for FMS, these options are yet hampered by relatively limited response rates versus placebo and frequent (and on occasion severe) side effects as well as the occurrence of tachyphylaxis [23]. In view of these limitations there is growing recognition regarding the importance of integrating nonpharmacological and complimentary approaches into the therapeutic strategy concerning FMS [24]. We will subsequently review existing data regarding the utilization of spa therapy for the treatment of FMS.

Buskila and colleagues [25] evaluated the effectiveness of balneotherapy on patients with FMS at the Dead Sea (Israel). This was a randomized prospective study of a 10-day treatment, including 48 patients randomized to sulfur bath (*n* = 24) or no treatment (*n* = 24). Relief in the severity of FMS-related symptoms (pain, fatigue, stiffness and anxiety) and reduced frequency of symptoms (headache, sleep problems and subjective swelling) were reported in both groups, but lasted longer in the treatment group. It was concluded that treatment of FMS at the Dead Sea was effective and safe. 

Neumann et al. [26] assessed the possible effect of balneotherapy at the Dead Sea area on the quality of life (QOL) of patients with FMS. It was found that staying at the Dead Sea spa, in addition to balneotherapy, can transiently improve the QOL of patients with FMS. The effect of combined Spa and physical therapy on pain in various chronic diseases including FMS was retrospectively studied in 472 spa and physical therapy patients [27]. It was demonstrated that a combination of Spa and physical therapy decreases pain and improves functional capacity without any hemodynamic risk in rheumatologic, neurological, and cardiac patients.

Zijlstra et al. [28] reported in a controlled randomized study that a combination of thalassotherapy (sea water therapy), exercise, and patient education can significantly improve symptoms and health-related quality of life in FMS. After 3–6 months, most outcome measures showed improvement. After 6 months, however, most differences between spa and control group were no longer statistically significant. The authors conclude that their combined program should be regarded as a palliative treatment with temporary effects. The cost effectiveness of an adjuvant treatment course of spa treatment compared with usual care only was estimated in FMS patients [29]. It was found that the temporary improvement in quality of life due to adjuvant treatment course of spa therapy for FMS patients is associated with limited incremental costs per patient.

Fioravanti et al. [30] provided a randomized clinical trial evaluating the effect and the tolerability of mud-bath treatment in FMS patients, who are poor responders to pharmacological therapy. Eighty FMS patients were randomly allocated to two groups: 40 were submitted to a cycle of 12 mud packs and thermal baths and 40 were considered as controls. Patients were evaluated by FIQ, tender points count, VAS for symptoms, AIMS 1, and HAQ. The results of this study showed the beneficial effects of a cycle of mud bath applications in FMS patients. All evaluation parameters significantly decreased at the end of the spa therapy cycle and remained stable after 16 weeks in comparison to baseline. The favorable effects extended to several domains including pain, fatigue, general health, and physical functioning.

 Jackson [7] updated the rheumatologic indications of spa therapy, based on clinical practice guidelines published by the French National Authority for Health (HAS) and the European League Against Rheumatism (EULAR) and on the results of randomized clinical trials. It was found that in FMS, EULAR recommends hot-water balneotherapy, an important component of spa therapy, rank B, based on 5 RCTS of which three were carried out in thermal springs. 

Sixteen studies indicated a persistent improvement (at least twelve weeks) in pain, analgesic and nonsteroidal anti-inflammatory drug consumption, functional capacity, and/or quality of life in several rheumatologic conditions including FMS.

It was concluded that spa therapy appears to be indicated for chronic low back pain, stabilized rheumatoid arthritis ankylosing spondylitis, and FMS [31].

 Falagas et al. [32] provided a systematic review on the therapeutic effect of balneotherapy. Twenty-nine RCTS were evaluated including six on FMS. It was concluded that there is a possibility that balneotherapy is associated with clinical improvement in rheumatologic disease mainly such as osteoarthritis, ankylosing spondylitis, rheumatoid arthritis, chronic low back pain, and FMS. 

Guidelli et al. [1] provided a review on spa therapy in FMS. They concluded that spa therapy seems to be effective and useful in FMS, reducing pain, improving function and ameliorating quality of life. In this review, possible mechanisms involved in the therapeutic effect of spa therapy in FMS have been enumerated. These include (but may not be limited to) a reduction in circulating levels of IL-1, PGE2, and LTB4 [33] an increase of serum malondialdehyde (MAD) and a decrease of serum superoxide dismutase (SOD) (possibly reducing oxidative stress) [34] as well as a combination of additional thermal, chemical, and mechanical factors [35].

### 5.1. Safety and Patient, Acceptability of Spa Therapy

Langhorst et al. [36], performing a systematic meta-analysis regarding the use of hydrotherapy for the management of FMS, have concluded that this form of treatment was associated with extremely unusual side effects and thus, not surprisingly, was highly acceptable for patients. In view of the side effects often associated with pharmacological treatment among FMS patients, this conclusion further underscores the practical utility of spa therapy among other complementary and nonpharmacological modalities, in the management of FMS. 

## 6. Conclusion

Spa therapy is an ancient and widespread practice, known to mankind and practiced by physicians over many centuries and in diverse cultures. While interest in this modality has waxed and waned and indications have evolved and changed, the persistent use of spa therapy for rheumatic (as well as dermatological) disorders and in particular for pain alleviation attests to the persistent efficacy of this treatment for these indications. This background underscores the applicability of spa therapy for the treatment of FMS, a chronic pain condition which may in fact overlap with much of what was previously described as “rheumatism”. In an age of growing recognition regarding the importance of natural and non-pharmacological approaches to chronic disorders, additional high-quality research is called for both into the optimal clinical utilization of spa therapy and into the mechanisms underlying this treatment in the management of FMS and chronic pain in general. Moreover, the potential role of spa therapy in the management of “secondary” FMS, that is, FMS coexisting with or overlapping with other conditions (inflammatory or otherwise) is also an area worthy of particular attention and research. 

Limitation inherent in the evidence-based approach to nonpharmacological modalities such as spa therapy must be acknowledged. It is inherently difficult to compare therapies which depend on local conditions such as climate, water composition, and atmospheric pressure. Thus, the possibility of making evidence-based recommendations applicable worldwide based on the results obtained at particular (and unique) sites is inevitably limited. This limitation must also be recognized in the narrative structure of the current review.

## Figures and Tables

**Table 1:** Examples of applications of Spa therapy over historical and geographical locations.

Condition/indications	Geographic location	Special characteristics
Restore strength, heal wounds	Ancient Greece Thermopiles, Kaifa	
Recuperation of wounded soldiers	Ancient Rome	
Psoriasis	Blue lagoon, Iceland	Geothermal activity
Dermatological disorders	Germany (Baltic area)	Sea water spas, PUVA-bath
Arthritis	Korea (Onyang holy spa)	
Psoriasis, psoriatic arthritis	Israel, Dead sea	High atmospheric pressure, extreme climate, and mud
